# Pharmacokinetics and pharmacodynamics of danaparoid during continuous venovenous hemofiltration: a pilot study

**DOI:** 10.1186/cc6119

**Published:** 2007-09-13

**Authors:** Anne-Cornélie JM de Pont, Jorrit-Jan H Hofstra, Derk R Pik, Joost CM Meijers, Marcus J Schultz

**Affiliations:** 1 Department of Intensive Care Medicine, Academic Medical Center, University of Amsterdam, Meibergdreef 9, 1105 AZ Amsterdam, The Netherlands; 2 Laboratory of Experimental Intensive Care and Anesthesiology, Academic Medical Center, University of Amsterdam, Meibergdreef 9, 1105 AZ Amsterdam, The Netherlands; 3 Faculty of Science, University of Leiden, Niels Bohrweg 1, 2333 CA Leiden, The Netherlands; 4 Laboratory of Experimental Vascular Medicine, Academic Medical Center, University of Amsterdam, Meibergdreef 9, 1105 AZ Amsterdam, The Netherlands

## Abstract

**Background:**

In patients with suspected heparin-induced thrombocytopenia (HIT) who need renal replacement therapy, a nonheparin anticoagulant has to be chosen to prevent thrombosis in the extracorporeal circuit. Danaparoid, a low-molecular-weight heparinoid consisting of heparan sulphate, dermatan sulphate, and chondroitin sulphate, is recommended for systemic anticoagulation in patients with HIT. However, there are few data on the use of danaparoid in patients with acute renal failure, especially in patients dependent on renal replacement therapy such as continuous venovenous hemofiltration (CVVH). In the present study, we analyzed the pharmacokinetics and pharmacodynamics of danaparoid during CVVH in patients with suspected HIT.

**Methods:**

Based on a mathematical model, a dosing scheme for danaparoid was designed, aiming at anti-Xa levels of 0.5 to 0.7 U/mL, with a maximum of 1.0 U/mL. This dosing scheme was prospectively tested in the first CVVH run of a cohort of five patients with suspected HIT. CVVH with a blood flow rate of 150 mL/minute and a substitution rate of 2,000 mL/hour was performed with a cellulose triacetate membrane. Danaparoid was administered as a continuous infusion of 100 anti-Xa-U/hour after a loading dose of 3,500 anti-Xa-U. Serial measurements of anti-Xa activity and prothrombin fragment F1+2 were performed at baseline, at *t *= 5, 15, and 30 minutes, and at *t *= 1, 2, 4, 8, 16, and 24 hours after the danaparoid loading dose.

**Results:**

The median anti-Xa activity reached a maximum of 1.02 (0.66 to 1.31) anti-Xa-U/mL after 15 minutes and gradually declined to 0.40 (0.15 to 0.58) anti-Xa-U/mL over the span of 24 hours. Target anti-Xa levels were reached from 2 to 12 hours after the loading dose. Median prothrombin fragment F1+2 gradually decreased from 432 (200 to 768) to 262 (248 to 317) pmol/L after 24 hours. No bleeding or thromboembolic events occurred throughout the described treatment period.

**Conclusion:**

Danaparoid administered by a continuous infusion of 100 anti-Xa-U/hour after a loading dose of 3,500 anti-Xa-U elicited target anti-Xa levels from 2 to 12 hours after the loading dose, without bleeding or thromboembolic events during the described CVVH treatment in patients with suspected HIT.

## Introduction

During continuous venovenous hemofiltration (CVVH), anticoagulation with unfractionated heparin is commonly used to prevent thrombosis in the extracorporeal circuit. However, in patients with suspected heparin-induced thrombocytopenia (HIT), another anticoagulant has to be chosen. Because patients with HIT have a 25% to 50% risk of symptomatic thrombosis, systemic anticoagulation is indicated [1]. In the American College of Chest Physicians guidelines for recognition, prevention, and treatment of HIT, direct thrombin inhibitors and danaparoid are recommended for systemic anticoagulation in patients with HIT [1]. Danaparoid is a low-molecular-weight heparinoid consisting of a mixture of heparan sulphate (84%), dermatan sulphate (12%), and small amounts of chondroitin sulphate (4%). Its pharmacological effect is exerted primarily by the inhibition of factors Xa and IIa at a ratio greater than that of unfractionated heparin [2].

Although extensive experience with danaparoid has been gained in the clinical setting, there are few data on its use in patients with acute renal failure, especially in patients dependent on CVVH. Under normal conditions, the elimination of danaparoid is predominantly renal, with an elimination half-life of 25 hours. During CVVH, danaparoid can be removed only by means of a polyarylethersulphone membrane, with a sieving coefficient of 0.78 ± 0.03 [3]. Therefore, treatment with a continuous infusion of danaparoid carries the risk of accumulation in patients with acute renal failure dependent on CVVH. Because an antidote is lacking, this accumulation may entail an increased risk of bleeding. The recommended dose for anticoagulation with danaparoid in HIT patients requiring CVVH is an intravenous (IV) loading dose of 2,250 anti-Xa-U followed by a continuous infusion of 600 anti-Xa-U/hour for the first 4 hours, 400 anti-Xa-U/hour for the next 4 hours, and then 200 to 400 anti-Xa-U/hour adjusted by anti-Xa level [4]. A therapeutic anti-Xa level is 0.5 to 0.7 anti-Xa-U/mL, with a maximum of 1.0 anti-Xa-U/mL. However, using the recommended dosing scheme, our patients frequently experienced bleeding, especially when peak anti-Xa levels exceeded 1.0 anti-Xa-U/mL. In a retrospective analysis, we found a linear relationship between the peak anti-Xa level and the need of red blood cell transfusions in patients with a peak anti-Xa level of greater than 0.7 anti-Xa-U/mL (*r*^2 ^= 0.55; *p *= 0.02) (ACJM de Pont, JJH Hofstra, DR Pik, JCM Meijers, MJ Schultz, unpublished data). Therefore, we decided to design a safer dosing scheme for danaparoid, based on a mathematical model aiming at a peak anti-Xa level of less than 1.0 and a maintenance level of between 0.5 and 0.7 anti-Xa-U/mL. Lindhoff-Last and colleagues [5] have suggested that a loading dose of 750 anti-Xa-U IV followed by a maintenance dose of 50 to 150 anti-Xa-U/hour might be sufficient to maintain a safe and effective level of anticoagulation. However, serial pharmacokinetic measurements to confirm this hypothesis have never been published. The aim of the present study was to determine the pharmacokinetic and pharmacodynamic properties of danaparoid in patients with suspected HIT treated with CVVH, using a new dosing scheme based on a mathematical model.

## Materials and methods

### Patients and study design

The observations in this study were made in the context of standardized protocol for routine patient care. Our institutional review board waived a formal approval procedure for the study. Eligible patients were suspected of HIT and had acute renal failure necessitating CVVH. Suspicion of HIT was based on the 4T score: (a) a more than 50% decrease in platelet count after exposure to heparin, (b) timing of the decrease in platelet count compatible with HIT, (c) a new thrombosis, skin necrosis, or an acute systemic reaction after heparin administration, and (d) absence of other causes of thrombocytopenia [6]. In addition, antibodies against heparin/platelet factor 4 (PF4) complex were detected by means of enzyme-linked immunosorbent assay (ELISA). The exclusion criterion was overt bleeding or a manifest clotting disorder defined as a prothrombin time or an activated partial thromboplastin time of more than 1.5 times the upper limit of normal. Enrolled patients were studied for the duration of the first CVVH run in which danaparoid was used as an anticoagulant.

### Hemofiltration procedure

Vascular access was obtained by insertion of a double-lumen catheter (Duo-Flow 400XL, 14F × 6 inches (15 cm); Medcomp, Harleysville, PA, USA) into a large vein (femoral, subclavian, or internal jugular vein). CVVH was performed using a Diapact hemofiltration machine (B. Braun Melsungen AG, 34212 Melsungen, Germany) and a cellulose triacetate hemofilter (CT-190G; Baxter Healthcare Corp., Deerfield, IL, USA). The ultrafiltration rate was set at 2,000 mL/hour, and a bicarbonate-buffered substitution fluid was administered in predilution mode with a flow of 2,000 mL/hour. The blood flow was set at 150 mL/minute, and a negative fluid balance was allowed. Circuit survival time was defined as the time elapsed from starting CVVH until clotting of the extracorporeal circuit.

### Mathematical model

Given a loading dose B (in anti-Xa-U) added to a total plasma volume of 3,500 mL and assuming an elimination half-life of 25 hours, the concentration at time point *t *will be 2^-*t*/25 ^times B/3,500 anti-Xa-U/mL. By adding a continuous dose D (in anti-Xa-U) per hour, the plasma concentration C(t) at time point *t *(in anti-Xa-U/mL) can be approximated by the formula

$$C(t)=\frac{1}{3,500}\left(B\alpha^{-t}+D\frac{1-\alpha^{-t}}{\alpha -1}\right),$$

where *α *= 2^1/25^. Ideally, the plasma concentration should be between 0.5 and 0.7 anti-Xa-U/mL. Thus, the value D is obtained by taking time to infinity, which yields *D *= 0.7 × 3.500 × (*α *- 1) anti-Xa-U, and consequently the loading dose B can be found as the maximal value for which the concentration does not exceed the value of 1.0 anti-Xa-U/mL.

### Anticoagulation

The extracorporeal circuit was not primed with any anticoagulant. Based on the mathematical model, the danaparoid loading dose B was calculated to be 3,500 anti-Xa-U and the continuous dose D to be 100 anti-Xa-U/hour. The CVVH procedure was started immediately after administration of the danaparoid loading dose of 3,500 anti-Xa-U followed by a continuous danaparoid infusion of 100 anti-Xa-U/hour.

### Blood collection, laboratory assays, and statistical analysis

Blood was collected in citrated vacutainer tubes at baseline, at *t *= 5, 15, and 30 minutes, and at *t *= 1, 2, 4, 8, 16, and 24 hours after the danaparoid loading dose and was processed immediately. Plasma was prepared by centrifugation at 2,500 *g *twice for 20 minutes at 16°C followed by storage at -80°C until assays were performed. Antibodies against heparin/PF4 complex were detected by ELISA (GTI PF4 HAT 45; Diagast, Loos, France). Anti-Xa activity was determined with Berichrom Heparin on a Behring Coagulation System (both from Dade Behring Marburg GmbH, Marburg, Germany). To assess the process of thrombin generation during CVVH, prothrombin fragments F1+2 (F1+2) were measured by ELISA (Enzygnost F1+2 [monoclonal]; Dade Behring Marburg GmbH). Normal values for F1+2 range from 300 to 1,600 pmol/L. Data are reported as median and range. Changes in coagulation parameters over time were compared by means of a paired Student's *t *test. Circuit survival times were compared with those previously published in the literature by means of Student's *t *test. A *p *value of less than 0.05 was considered significant.

## Results

### Patient characteristics

Five critically ill patients with acute renal failure and suspicion of HIT were studied. All patients had a previous exposure to heparin in the past 30 days, and in all patients the platelet count decreased more than 50% within 1 day after rechallenge. Two patients suffered from skin necrosis, and in no patient could a definite alternative cause for the thrombocytopenia be found. For all patients, 4T scores were calculated; these are summarized in Table 1. All patients had a 4T score compatible with an intermediate (4 to 5) or high (6 to 8) probability of HIT. With the exception of patient 2, all patients had positive antibodies against the heparin/PF4 complex.

**Table 1:** Characteristics of the enrolled patients

Patient	Gender	Age (years)	Body weight (kg)	Diagnosis	Etiology of ARF	Type of ARF	APACHE II score	4T score	Circuit survival time (hours)
1	Male	76	75	Ventricular rupture	Cardiac failure	Nonoliguric	23	4	62.3
2	Female	76	72	Post-CABG × 5	Cardiac failure	Oliguric	20	8	50.2
3	Female	71	62	Rectal resection	Sepsis	Oliguric	24	4	20.0
4	Female	65	70	Post-CABG × 3	Cardiac failure	Oliguric	21	8	89.0
5	Male	75	70	Post-CABG × 5	Cardiac failure	Oliguric	20	5	29.3

4T score, probability score for heparin-induced thrombocytopenia, based on extent, timing, and cause of thrombocytopenia and complications of heparin administration; APACHE II, Acute Physiology And Chronic Health Evaluation II; ARF, acute renal failure; CABG, coronary artery bypass grafting.

### Pharmacokinetics and pharmacodynamics of danaparoid during continuous venovenous hemofiltration

**Figure 1:**
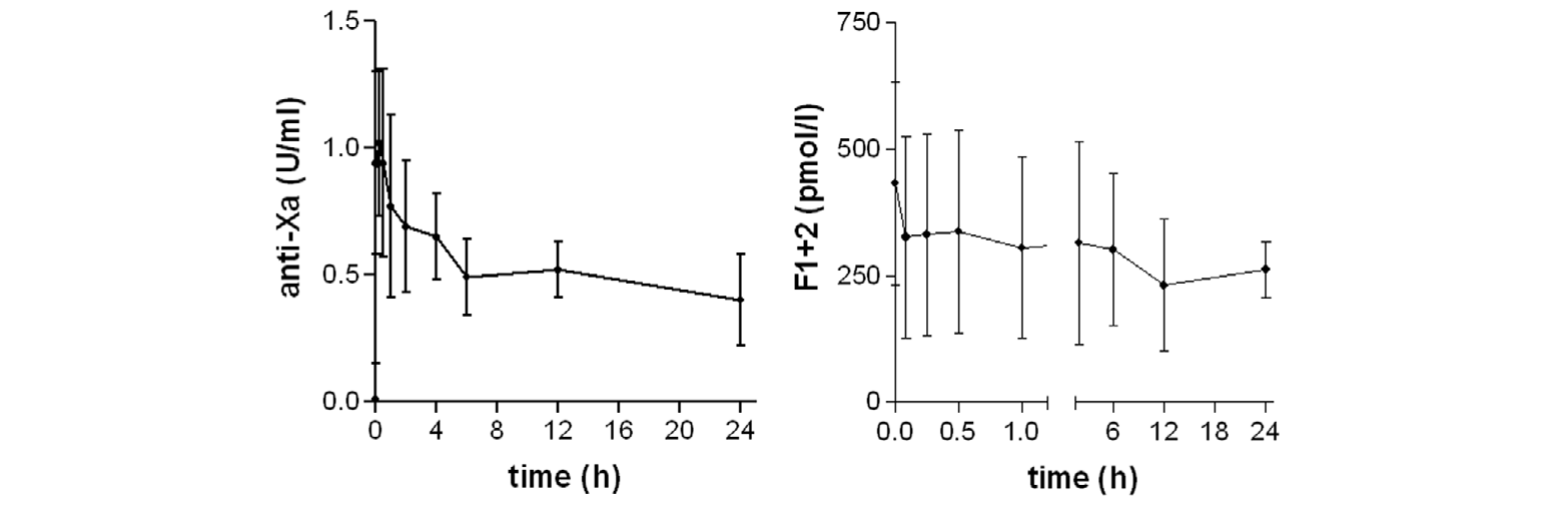
Pharmacokinetics and pharmacodynamics of danaparoid during continuous infusion after a loading dose. Course of the levels of anti-Xa activity (left panel) and prothrombin fragment F1+2 (right panel) during the first 24 hours of treatment with a continuous danaparoid infusion of 100 anti-Xa-U/hour after a loading dose of 3,500 anti-Xa-U. Data represent median and range.

Median anti-Xa activity reached a maximum of 1.02 (0.66 to 1.31) U/mL at *t *= 15 minutes (*p *= 0.001 compared with *t *= 0) and gradually declined to 0.40 (0.15 to 0.58) U/mL over the span of 24 hours (*p *< 0.05 compared with *t *= 15 minutes). The half-life of the anticoagulant effect as calculated from these data was 8 hours. Mean prothrombin fragment F1+2 decreased from 432 (200 to 768) to 326 (131 to 697) pmol/L at *t *= 5 minutes (*p *< 0.05) and did not change significantly thereafter (Figure 1).

### Complications

No clinically important bleeding events or thromboembolic complications occurred in any of the five patients during the described CVVH treatment.

### Circuit survival times

The individual circuit survival times reached with danaparoid as an anticoagulant during CVVH are reported in Table 1. A median circuit survival time of 50.2 (20 to 89) hours was achieved.

## Discussion

In this small prospective cohort study, we demonstrated that using danaparoid in a loading dose of 3,500 anti-Xa-U IV followed by a continuous infusion of 100 anti-Xa-U/hour, the median peak anti-Xa level reached was slightly too high, whereas the median anti-Xa level fell below the target range of 0.5 to 0.7 U/mL after 12 hours. Thrombin generation remained within the normal range during the first 6 hours. To our knowledge, this is the first time the pharmacokinetics and pharmacodynamics of danaparoid have been studied during CVVH. A limitation of this study is that we used an ultrafiltration rate of 2 L/hour, which is lower than the 35 mL/kg per hour proven by Ronco and colleagues [7] to be most effective. Additional studies are needed to determine the optimal danaparoid dosing scheme during CVVH with an ultrafiltration rate of 35 mL/kg per hour or more.

Although in our cohort the median anti-Xa activity dropped below 0.5 U/mL after 12 hours, we achieved median circuit survival times similar to those reported by Lindhoff-Last and colleagues [5]: 50.2 (20 to 89) hours versus 36 (24 to 70) hours (*p *value not significant). However, their mean circuit survival time was achieved with a lower loading dose (or no loading dose at all) followed by a continuous infusion varying from 90 to 225 anti-Xa-U/hour, reaching a mean anti-Xa activity varying from 0.33 to 0.89 U/mL. In addition, Lindhoff-Last and colleagues reported that with continuous venovenous hemodialysis, an even lower dose of danaparoid was effective: with a loading dose of 750 anti-Xa-U IV followed by a continuous infusion varying from 64 ± 10 to 315 ± 163 anti-Xa-U/hour, an anti-Xa activity of 0.23 ± 0.13 to 0.53 ± 0.17 U/mL was reached. Unfortunately, the circuit survival times achieved with this dose were not reported. However, in a study on anticoagulation with low-molecular-weight heparins during CVVH, we did not find a relationship between anti-Xa activity and circuit survival time: with a maximum anti-Xa activity of 0.46 ± 0.14 U/mL gradually declining over the span of 24 hours, a circuit survival time of 15.4 ± 7.4 hours was reached [8]. This finding confirmed an earlier finding by Journois and colleagues [9], who did not find a relationship between anti-Xa levels and circuit survival times either. A recent randomized controlled crossover study among 40 critically ill patients also failed to establish a correlation between anti-Xa levels and filter survival [10].

Because bleeding complications are related to the anti-Xa activity reached [11], it is important to use the lowest possible dose of danaparoid that is still effective during CVVH. As can be calculated by our proposed formula, this might be achieved by lowering the loading dose, the level of continuous infusion, or both. Given that a loading dose of 3,500 anti-Xa-U IV led to a median maximum anti-Xa activity of 1.02 (0.66 to 1.31) U/mL, lowering the loading dose is recommended. Continuous infusion of 100 IU/hour was effective, as anti-Xa activities remained within the target range during the first 12 hours, leading to acceptable circuit survival times. Additional studies are needed to determine the lowest danaparoid dose for both loading and continuous infusion necessary to keep the circuit open.

## Conclusion

This study demonstrated that danaparoid in a loading dose of 3,500 IU IV followed by a continuous infusion of 100 IU/hour was effective at keeping the extracorporeal circuit open, with median anti-Xa activities within the therapeutic range from 2 to 12 hours after the loading dose and without any bleeding or thomboembolic complications during the described treatment period.

## Key messages

• When danaparoid was used as an anticoagulant during continuous venovenous hemofiltration, a loading dose of 3,500 IU IV followed by a continuous infusion of 100 IU/hour led to target anti-Xa levels of 0.5 to 0.7 U/mL from 2 to 12 hours after the loading dose.

• To reach a peak anti-Xa level within the target range, the loading dose should be lowered according to the mathematical formula, aiming at a peak anti-Xa level of 0.5 to 0.7 U/mL.

• When danaparoid was administered as a continuous infusion of 100 IU/hour after a loading dose of 3,500 IU, a median circuit survival time of 50.2 hours was reached, while no clinically important bleeding events or thromboembolic complications occurred during the particular hemofiltration run.

## Abbreviations

CVVH = continuous venovenous hemofiltration; ELISA = enzyme-linked immunosorbent assay; F1+2 = prothrombin fragment F1+2; HIT = heparin-induced thrombocytopenia; IV = intravenous; PF4 = platelet factor 4.

## Competing interests

MS received a €30,000 grant from Organon International Inc. (Roseland, NJ, USA) as a contribution to a randomized controlled clinical trial comparing two danaparoid dosage schemes with standard heparin during continuous venovenous hemofiltration. This trial was scheduled to be performed in the second half of 2007. The present manuscript was not financed by Organon International Inc. The other authors declare that they have no competing interests.

## Authors' contributions

ACdP designed the study and treated the patients. DP designed the mathematical model. JM was responsible for the performance of the laboratory assays. JJH was responsible for the analysis of the data. MS supervised the study. All authors contributed in the writing and critical appraisal of the manuscript, and all authors read and approved the final manuscript.
